# Effects of reproductive resource allocation and pollen density on fertilization success in plants

**DOI:** 10.1186/s12898-020-00290-x

**Published:** 2020-05-02

**Authors:** Elizabeth M. Gillet, Hans-Rolf Gregorius

**Affiliations:** 1 Forstgenetik und Forstpflanzenzüchtung. Fakultät Forstwissenschaften und Waldökologie, Universität Göttingen, Büsgenweg 2, 37077 Göttingen, Germany; 2 Institut für ökologische und Populationsgenetik (IPOEG), Am Pfingstanger 58, 37075 Göttingen, Germany

**Keywords:** Climate change, Gamete production, Mathematical model, Ovule fertilization, Pollen dispersal, Pollen limitation, Resource decline, Reproduction

## Abstract

**Background:**

Declining resources due to climate change may endanger the persistence of populations by reducing fecundity and thus population fitness via effects on gamete production. The optimal mode of generative reproduction allocates the limited resources to ovule and pollen production in proportions that maximize the number of fertilized ovules in the population. In order to locate this optimum and derive reproduction modes that compensate for declined resources to maintain reproductive success, a model of gamete production, pollen dispersal, and ovule fertilization is developed. Specification of opportunities for compensation is given priority over specification of physiological or evolutionary mechanisms of adaptation. Thus model parameters summarize gametic production resources, resource investment per gamete, resource allocation as proportion of resources invested in ovules, and pollen density as size of the pollen dispersal range and proportion of pollen retained within the range. Retained pollen disperses randomly, and an ovule is fertilized if at least one pollen settles on its surface. The outcome is the expected number of fertilized ovules.

**Results:**

Maximization of fertilization success is found to require the investment of more gametic production resources in ovules than in pollen, irrespective of the parameter values. Resource decline can be compensated by adjusting the resource allocation if the maximum expected number of fertilized ovules after the decline is not less than the expected number the population experienced before the decline. Compensation is also possible under some conditions by increasing the pollen density, either by raising a low pollen retention or by shrinking the dispersal range.

**Conclusion:**

Fertilization success in populations affected by resource decline may be maintainable by adjustment of the sexual allocation of gametic production resources or by increasing pollen density. The results have implications for insect pollination, sexual allocation bias, management measures, and metapopulation fragmentation.

## Background

The persistence of a population critically depends on the success of its system of reproduction in producing offspring to offset mortality. For a generatively reproducing species, the response of populations must be to produce sufficient numbers of ovules and pollen in proportions that ensure the fertilization of as many ovules as possible and their development into seeds. For a cosexual plant species, each individual typically allocates its resources to both gametic functions in that it produces a number of ovules and many times this number of pollen. Disregarding individual differences (see review of Barrett and Harder [2]), the reproductive success of the population as a whole depends on the total number of pollen and ovules produced within the population.

In the face of the currently pressing problem of climate change, the resources provided by the environment may decline. Since the resources available for reproduction place limits on gamete production, populations cannot produce arbitrarily large numbers of gametes to ensure reproductive success [1, 6]. In effect, limited resources may impose a trade-off between the numbers of pollen and ovules that can be produced. Production of a large number of ovules provides the potential for a large number of seeds, but only if the number of pollen is sufficient to ensure that most of the ovules are actually fertilized. Since a large proportion of pollen do not settle in the vicinity of ovules, especially in wind-pollinated plants, plants typically produce many more pollen than ovules. Because the investment of resources per ovule is usually higher than that per pollen, the production of one less ovule frees resources for the production of several pollen. The optimal mode of reproduction of a population would thus be to allocate the available resources to ovule and pollen production in proportions that serve to maximize the number of fertilized ovules for development into seeds (as an elementary component of population fitness) under the given pattern of pollen dispersal. The term mode of reproduction as used here comprises gamete production, zygote formation, and seed development (e.g. in the sense of Fryxell [14]).

Basic conditions for reproduction are set by the environment. Not only do meteorological conditions influence the dispersal of pollen to the ovules, whether by wind or insects. The environment also determines the availability of resources for both phases of reproduction, the generative phase beginning with gamete formation and ending with the fertilized ovule (zygote), and the subsequent vegetative phase during which the zygote develops into a seed. During the generative phase, resources must be additionally allocated to the two sexual functions, i.e., to the production of pollen and ovules. Here we focus on the generative phase as the defining characteristic of populations as reproductive communities.

For populations that have adapted their modes of reproduction to a relatively stable environment, changes in the environmental conditions can lead to fewer fertilized ovules, for example through reallocation of resources from reproduction to vegetative growth [3]. Confronted with environmental changes that would reduce population fitness if the population retains its current mode of reproduction, the question is whether, and if so under what conditions, the projected fitness loss of the population could be compensated (i.e., its current fitness maintained) by adjustment of its reproduction parameters. Adjustment can take place in two ways, either physiologically (via acclimatization) or evolutionarily (via genetic selection over generations) [4]. Both ways can equally affect fertilization success.

The question of whether compensation is possible is embedded in the wider problem of recognizing environmental conditions that are so severe that the adaptive potential is not broad enough to maintain the previous level of reproductive success. Here the term adaptive potential is applied to describe a population’s ability to respond to environmental changes by means of physiological or evolutionary adjustment of its reproduction parameters (in the sense of Eizaguirre and Baltazar-Soares [10]). Expressed in more mathematical terms, the adaptive potential is limited by the combined set of realizable ranges of the parameters of the reproduction system. Thus the question whether the adaptive potential of a population enables compensation in the face of environmental deterioration is closely related to delimitation of the set of environmental conditions to which this population can adapt. Evaluation of the adaptive potential of species in connection with the severity of environmental changes is of special current interest in the face of climate change (see e.g. reviews of Franks and Hoffman [13] and Kelly [19]). In forestry, for example, it is being debated whether native tree species will be able to adapt to the rapidly changing environmental conditions or whether it is advisable to introduce “exotic” species that are already adapted to the predicted future conditions.

Here we model the number of fertilized ovules that are produced within an entire population under a mode of reproduction that is specified by a constellation of parameters realizable within the limits set by the adaptive potential, without regard for the variability of the contributions of its individuals. In order to concentrate on the problem of whether compensation for loss of population fitness is at all possible, we reduce the complexity of the reproduction system to a small number of parameters, each of which summarizes the output of highly complex mechanisms. Only limitations to population fitness (in terms of fertilized ovules) set by the adaptive potential are analyzed. Mechanisms for adjustment of the reproduction mode within the population, whether physiological or evolutionary, are not dealt with specifically, but examples are suggested. Especially the mechanisms of evolutionary adaptation may require more detailed treatment, since they involve assumptions on the inheritance of the mode of reproduction (parameters) that may guarantee establishment in the sense of a game theoretic strategy but not population survival (see e.g. Spencer and Feldman [24]). In any case, if the change in environmental conditions is so severe that compensation via adjustment of the admissible parameter values is not possible, then neither physiological nor evolutionary mechanisms whatsoever can succeed. For this reason, the present paper focuses on the opportunities for compensation by changes in parameters, and not on specific physiological or evolutionary mechanisms of adaptation.

### Objectives

While it is sometimes possible to estimate the number of seeds produced in a habitat of interest, it is much more difficult to infer the reproduction parameters underlying their generation. Among these are the total number of gametes (pollen and ovules), the pattern of pollen dispersal, and the proportion of the ovules that were fertilized and became seeds. Perhaps for this reason, many studies on wind-pollinated trees assume that pollen is available in sufficient numbers to fertilize all ovules, despite observations of pollen limitation (see e.g. Guo et al. [17]). From this assumption, it follows that the number of fertilized ovules is the same as the number of ovules produced by the individuals and that the number of pollen is effectively infinite. Not only in degraded or fragmented habitats, however, is it conceivable that reduced pollen production in connection with an inadequate dispersal pattern leaves ovules unfertilized. This is especially true for wind-pollinated species, where a large portion of the pollen may settle in places where there are no ovules and thus be unavailable for reproduction. It may also be true for insect-pollinated species when considering the increase in pollen loss with the distance between ovules and the various forms of pollen consumption, including export to hives, but the complex issues of insect pollination will not be explicitly treated here.

Modes of reproduction that compensate for a reduction in gametic production resources in order to maintain the reproductive success of a population are sought here with the help of an elementary probabilistic model of reproduction that is sequentially structured into three modules: gamete production, pollen dispersal, and ovule fertilization. Of the various ecological and evolutionary factors that play a role in determining how many ovules are fertilized, two are investigated here: sexual allocation as the proportion of gametic production resources devoted to the two gametic sexes, and pollen density.

The model is based on a number of input parameters. Three of them directly concern the resources, namely the total resources available for gamete production and the resource investment per ovule and per pollen. The sexual allocation parameter specifies the proportion of resources invested in ovules, the remainder being invested in pollen, from which the numbers of ovules and pollen are calculated. A given proportion of the produced pollen is assumed to disperse randomly (i.e., uniformly) over a dispersal range containing the ovules, with the remainder drifting beyond the bounds of the range. This specifies the pollen density within the dispersal range (i.e., the mean number of pollen per unit area) as a value that can be estimated in real populations. Ovules are assumed to be fertilized if a specified minimum number of pollen settle on their pollen-catching devices (anatomical structures vary widely between species: in angiosperms the stigma, in some conifers the pollination drop). Due to the randomness of pollen dispersal, the smaller the range across which pollen-catching devices are effective (called surface), the fewer pollen are expected to settle on it and be available for its fertilization. For probability theoretical convenience, the size of the dispersal range is thus measured in units equal to the average size of the pollen-catching surface of ovules (or rather its projection onto the plane).

The main outcome of the model is the expected number of fertilized ovules produced by the population. This expectation is simply the product of the number of ovules and the expected proportion of ovules that are fertilized, which is in turn a function of the amount of resources, the resource investment per gamete, the sexual allocation, and the size of the dispersal range and the proportion of pollen it retains. Since these parameters may change over time, the expected number of fertilized ovules may always be less than the maximum number that could be reached for the momentary values of the parameters.

The main objectives are to determine the optimal allocation of gametic production resources that maximizes the fertilization success of the population as well as to derive modes of reproduction that compensate for resource reduction and detect their limitations. The investigated reproduction modes include adjustment of the sexual allocation of the resources and the pollen density. The results are obtained analytically and demonstrated graphically for different sets of parameter values.

## Methods

Generative reproduction leading up to the fertilization of ovules consists of three major processes that operate sequentially: gamete production, pollen dispersal, and fertilization (gametic fusion). Each is modeled as a module and then combined to yield the expected number of fertilized ovules $E(F^*)$.

### The model

#### Gamete production module

The task of this module is to determine the numbers *F* of ovules and *M* of pollen that are produced by the population (notation is summarized in Table 1). It is based on the assumption that the total resources available for gamete production are limited to an amount *R* and that the investment of resources per gamete is fixed at $r_f$ for one ovule and $r_m$ for one pollen. This corresponds to the specification of resource investment by Queller [21] in quantification of the relationship between the pollen-ovule ratio as numbers and the investment of resources to male and female function. As a rule, the amount of resources invested in the production of one pollen is less than in one ovule, i.e., $r_m<r_f$.

**Table 1:** Model parameters. Constraints between parameters are specified in the text

Gamete production model	
*R* = Total resources used for gamete production, measured e.g. in units of energy	
$r_f$ = Resource investment for production of one ovule in the same units as *R* (*f* for female)	
$r_m$ = Resource investment for production of one pollen in the same units as *R* (*m* for male)	
$\alpha$ = Sexual allocation of resources to ovules as the proportion of *R* that is used to produce ovules ($\alpha {\in }(0,1)$). The remainder $(1{-}\alpha ){\cdot }R$ is used to produce pollen	

Outcome of the gamete production model	
*F* = Number of ovules produced	
*M* = Number of pollen produced	

Ovule fertilization model	
*h* = Size of the pollen dispersal range expressed as the number of pollen-catching surfaces of an ovule that can be projected onto this range	
*w* = Pollen retention as the proportion of the *M* pollen that remain within the pollen dispersal range	
*d* = Pollen density *wM*/*h* within the pollen dispersal range	

Outcome of the ovule fertilization model	
*E*(*P*) = Expected proportion of fertilized ovules	
$E(F^*)$ = Expected number of fertilized ovules	

For given *R*, $r_f$, and $r_m$, and assuming that all of the resources *R* are invested in gamete production, the numbers of ovules *F* and pollen *M* are mutually constrained by the equation

1 $$\begin{aligned} R\, =\,{r_f} \cdot F+{r_m}\cdot M \end{aligned}$$

Thus the number of gametes of one sex can be expressed as a function of the given number of gametes of the other sex by the linear relationships

$$\begin{aligned} F\, =\, & {} (R{-}r_m\cdot M)/r_f\\ M=\, & {} (R{-}r_f\cdot F)/r_m \end{aligned}$$

As *F* increases from 0 to its maximum of $R/r_f$, *M* decreases from its maximum of $R/r_m$ to 0.

Define the *sexual allocation*$\alpha {\in }(0,1)$ as the proportion of the resources *R* that are invested in the production of ovules. Then the numbers *F* of ovules and *M* of pollen equal

$$\begin{aligned} F\, =\, & {} \alpha {\cdot }R/r_f\\ M=\, & {} (1{-}\alpha ){\cdot }R/r_m \end{aligned}$$

When $\alpha$ is small, most of the resources are used for pollen production; when $\alpha$ is large, most of the resources are devoted to ovule production. For $\alpha = 0.5$, half of the maximum number $R/r_f$ of ovules and half of the maximum number $R/r_m$ of pollen are produced.

This definition of sexual allocation as a proportion of resources (also see [15]) is preferred here over the more commonly used pollen-ovule ratio *P*/*O*, i.e., the number of pollen per ovule (see [8, 20]), because the notion of allocation as a proportion reflects the realistic assumption that resources are limited. Besides, *P*/*O* approaches $\infty$ when *O* approaches 0. The question whether the pollen-ovule ratio *P*/*O* in flowering plant species correlates with other characteristics of the mating system has been intensively studied. Based on large numbers of species, interspecific correlation of *P*/*O* with factors such as pollen size [5], pollen number [15], or the efficiency of pollination as determined by the breeding system [8, 12] was studied. Some studies find correlations and some do not.

Note that by considering the relative investment $r_m/r_f$ of resources in single pollen per single ovule as a second pollen-ovule ratio, the sexual allocation $\alpha$ can be expressed for all $F{>}0$ solely as a function of the two pollen-ovule ratios *M*/*F* and $r_m/r_f$ as

$$\begin{aligned} \alpha = \frac{1}{1+\displaystyle \frac{r_m}{r_f}\cdot \frac{M}{F}} \end{aligned}$$

#### Pollen dispersal module

The outcomes *F* and *M* of the gamete production module set limits on the number $F^*$ of fertilized ovules, the most obvious being $F^*\le \min \{F,M\}$. In order for a single pollen to have the chance to fertilize, it must settle on the pollen-catching surface of an ovule. The pollen produced within the population are assumed here to disperse over a bounded *pollen dispersal range* that encompasses the population and thus contains all of the *F* ovules. The exact positions of the ovules within this range are not specified.

Following the model of Gregorius [16], let the pollen-catching surface of an ovule (e.g. stigmatic area [9]) be of average area *z*. Partitioning the pollen dispersal range into *h* spatial units of size *z* yields an expression for its size in terms of the size of pollen-catching surfaces (see bottom of Table 1). Assume that no more than one ovule can occupy a spatial unit and that all of the produced ovules lie within the pollen dispersal range, from which it follows that $h{\ge }F$.

Assume that a proportion $w{\in }(0,1)$ of the *M* pollen remains within the range. This implies that the proportion $(1{-}w){\cdot }M$ is lost for purposes of fertilization. Assume further that the *wM* pollen disperse at random over the *h* spatial units, following a uniform distribution of pollen over the dispersal range. This implies that any number of pollen can settle within any of the *h* spatial units, including zero, and that many pollen may settle in places where there is no ovule. The assumption of random pollen dispersal gives this module the characteristic of a reference scenario for comparison of the outcome with more complex forms of pollen dispersal, such as distance-dependence, which are difficult to ascertain experimentally.

Define the *pollen densityd* as the mean number of pollen that settle within each of the *h* spatial units of the pollen dispersal range. Due to the assumption of random pollen dispersal, *d* equals the ratio *wM*/*h* of the total number of pollen to the number of spatial units. Since *d* does not depend on how many of the *h* spatial units actually hold an ovule, *d* equals the mean number of pollen that settle on the pollen-catching surface of any ovule.

#### Ovule fertilization module

**Fig. 1:**
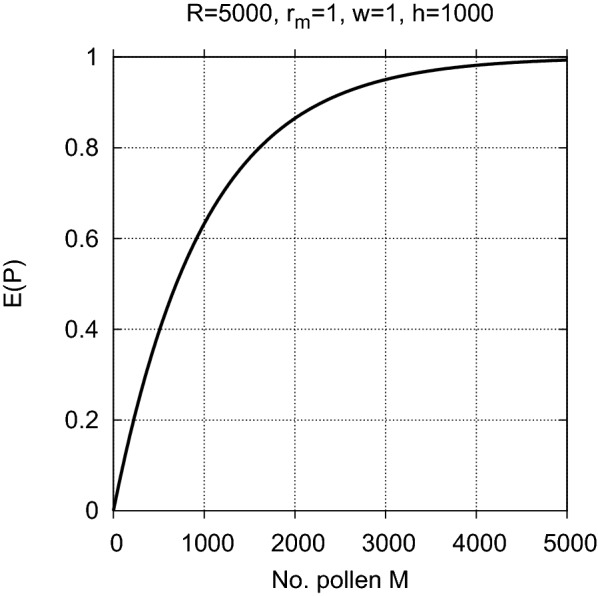
The expected proportion *E*(*P*) of fertilized ovules as a function of the number *M* of pollen ranging from 0 to its maximum of $R/r_m = 5000$ (for $R = 5000$ and $r_m = 1$), all of which ($w = 1$) settle within the dispersal range of size $h = 1000$. *E*(*P*) is close to 0 if there are few pollen and increases towards 1 as *M* approaches its maximum of $R/r_m$. It is independent of the number of ovules

Assume that an ovule is fertilized if at least one pollen settles within its spatial unit, i.e., on its pollen-catching surface. Following the model of Gregorius [16], where *P* denotes the random variable describing the proportion of ovules that are fertilized, the *expected proportionE*(*P*) *of fertilized ovules* among all ovules equals

$$\begin{aligned} E(P)=1-\left( 1{-}\frac{1}{h}\right) ^{wM} \end{aligned}$$

which is the binomial probability that at least one pollen settles within any given spatial unit, whether it contains an ovule or not. It is calculated as one minus the probability that all of the *wM* pollen land in the other $h{-}1$ spatial units. *E*(*P*) is an increasing function of *M* and thus a decreasing function of *F* (see Fig. 1).

The variance of *P* is a measure of the reliability of the expected proportion as an estimate. Gregorius [16] calculates the variance as

$$\begin{aligned} V(P)= & {} \left( 1{-}\frac{1}{F}\right) \cdot \left( 1{-}\frac{2}{h}\right) ^{wM} -\left( 1{-}\frac{1}{h}\right) ^{wM}\cdot \left[ \left( 1{-}\frac{1}{h}\right) ^{wM} -\frac{1}{F}\right] \\= & {} \frac{1}{F}\cdot \left[ \left( 1{-}\frac{1}{h}\right) ^{wM} -\left( 1{-}\frac{2}{h}\right) ^{wM}\right] -\left[ \left( 1{-}\frac{1}{h}\right) ^{2wM} -\left( 1{-}\frac{2}{h}\right) ^{wM}\right] \end{aligned}$$

(A typographical error in the original publication is corrected here).

Due to the uniform distribution of the pollen over the dispersal range, the fertilization probability of any given ovule does not depend on its position within the range. Thus the actual distribution of the ovules over the *h* spatial units plays no role in this module. Together with the assumption of random pollen dispersal, this allows the total gamete production of the population to be viewed collectively, thus avoiding the need to specify the number of individuals or the numbers of gametes that each individual produces.

#### Model synthesis

To determine the main outcome of the model, recall that each of the *F* ovules occupies one of the *h* spatial units into which the pollen dispersal range is partitioned (requiring $F{\le }h$) and that the proportion *w* of all produced pollen is retained within the dispersal range, i.e., the number $wM = w{\cdot }(R{-}r_f{\cdot }F)/r_m$. The expectation $E(F^*)$ for the number $F^*$ of fertilized ovules then equals the number *F* of ovules times the expected proportion *E*(*P*) of ovules that are fertilized, that is, the *expected number of fertilized ovules* equals

$$\begin{aligned} E(F^*)=F\cdot E(P) =F\cdot \left( 1{-}\left( 1{-}\frac{1}{h}\right) ^{wM}\right) \end{aligned}$$

**Fig. 2:**
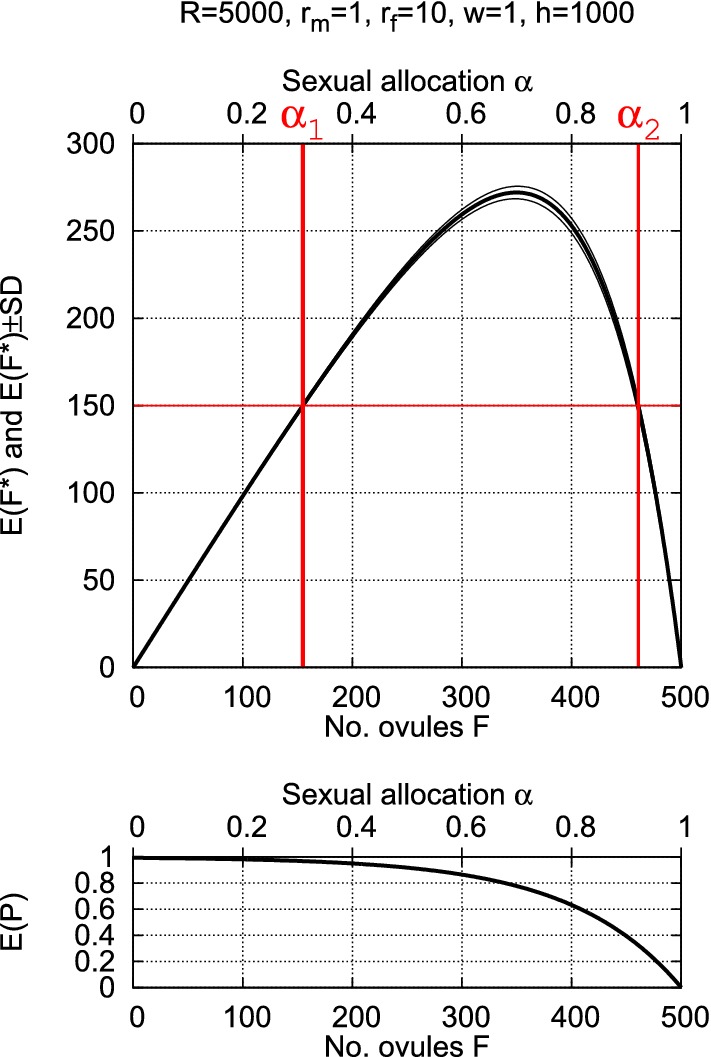
The expected number $E(F^*)$ of fertilized ovules $F^*$ (upper panel, framed by the standard deviation *SD* of $F^*$) is the product of the number of ovules *F* (lower x-axis) and the expected proportion of fertilized ovules $E(P) = \left( 1{-}\frac{1}{h}\right) ^{wM}$ (lower panel), where $M=(R{-}r_f{\cdot }F)/r_m$ is the number of pollen produced when *F* ovules are produced. The model parameters equal $R = 5000$, $r_f = 10$, $r_m = 1$, $w{=}1$, and $h = 1000$ (for notation see Table 1). The scaling of the upper x-axis shows the sexual allocation $\alpha$ corresponding to *F* on the lower x-axis. For both allocations $\alpha _1$ and $\alpha _2$, the expected number of fertilized ovules equals 150 (red lines)

Both $E(F^*)$ and the underlying *E*(*P*) are illustrated in Fig. 2.

In terms of the model parameters *R*, $r_f$, $r_m$, *w*, *h*, and $\alpha$, for which $F=\alpha R/r_f$ and $M=(1{-}\alpha ){\cdot }R/r_m$, the expected number $E(F^*)$ of fertilized ovules equals

$$\begin{aligned} E(F^*) =F{\cdot }E(P) =\frac{\alpha R}{r_f}{\cdot }\left( 1-\left( 1{-}\frac{1}{h}\right) ^{(1{-}\alpha ){\cdot }wR/r_m}\right) \end{aligned}$$

Defining the compound variable

$$\begin{aligned} b=\left( 1{-}\frac{1}{h}\right) ^{wR/r_m} \end{aligned}$$

these expressions can be simplified to

$$\begin{aligned} E(P)=1{-}b^{1{-}\alpha } \qquad and\qquad E(F^*) =\frac{\alpha R}{r_f}{\cdot }\left( 1{-}b^{1{-}\alpha }\right) \end{aligned}$$

Note that *b* equals the probability that a particular spatial unit remains empty if a proportion *w* of the resources were allocated to the production of pollen. Hence, $b^{1{-}\alpha }$ equals the probability of a particular spatial unit to remain empty if a proportion $w(1{-}\alpha )$ of the resources were allocated to the production of pollen. In this interpretation of *b*, the retention of pollen is translated into the proportion of resources allocated to production of pollen that remains in the range.

### Determinants of the model parameters

Each of the model parameters summarizes highly complex conditions and processes that can be subject to variation. The gametic production resources *R* that are available to the population (quality and quantity) are determined by and can vary with the environmental conditions. These conditions include factors that are temporally relatively stable, such as soil type and nutrient availability, and annually varying factors, such as temperature and precipitation.

The resource investments $r_m$ and $r_f$ per gamete, in contrast, are more likely to be genetic traits. These should depend on the complexity of the forms of the gametes, with larger or more elaborately constructed gametes being more “expensive”. Changes in $r_m$ or $r_f$ should go along with genetic, especially evolutionary, changes in gamete construction.

Sexual allocation can be viewed as a parameter that is affected by both genetics and the environment. It may be a genetically controlled physiological reaction to annually varying environmental conditions (temperature, moisture). Individual pollen-ovule ratios are found to vary considerably within the same population (sexual asymmetry) and between years in numerous plant species, including trees (see review of Ross [23]). Whereas the sexual allocation of resources may be sensitive to environmental variation between years, the range within which the allocation varies may also be a fixed genetic trait that is characteristic of the sexual system of the species and the number and fecundity of individuals of each genetic type (the most obvious sexual system being dioecy).

The size *h* of the pollen dispersal range is also dependent on environmental conditions, especially the velocity, direction, and turbulence of the wind for wind-pollinated species. Changes in the climatic conditions can lead to stronger winds or higher updrafts that increase the meso-scale dispersal of pollen [25], reducing pollen retention *w* within the range. *h* also depends on topography and the structure and height of the surrounding vegetation, where barriers to wind-borne pollen dispersal may arise at the boundaries of the previous range, restricting pollen flow to a smaller dispersal range. Insect pollination is more complex, since insects can transport pollen directly to ovules or they can transport pollen outside of the range (e.g. to their hives), and self-fertilization is even more of an issue than for wind-pollinated species. The underlying size $z{=}1/h$ of the pollen-catching surface of an ovule can vary with the investment $r_f$. Note that here it is assumed that each pollen-catching device holds a single ovule, requiring adaptation of the model for species that have multiple ovules at the end of a stigma.

## Results

### Optimizing sexual allocation

The optimal sexual allocation would yield the largest expected number of fertilized ovules $E(F^*)$ for the given model parameters. In numerous model-based studies of dioecious species, including plants, the optimal sex ratio of female to male individuals was determined to be greater than one, meaning that maximization of the number of fertilized ovules requires an excess of females [16]. Considering that the proportion of female and male individuals in a dioecious species is analogous to the sexual allocation of resources of the cosexual individuals of a monoecious species to ovule and pollen production, this phenomenon carries over to the present model.

The optimal sexual allocation possesses three important properties in this model (see “Appendix”).

- For any given set of model parameters *R*, $r_f$, $r_m$, *w*, *h* there exists a unique optimal allocation $\hat{\alpha }$ that maximizes the expected number of fertilized ovules $E(F^*)$, and it is implicitly expressed by the equation $b^{1{-}\hat{\alpha }}\left( \hat{\alpha }\ln (b)-1\right) +1=0$, for the variable $b = \left( 1{-}\frac{1}{h}\right) ^{wR/r_m}$ defined above (Proposition 1). No closed-form solution of this implicit expression for $\hat{\alpha }$ could be found.
- The optimal allocation devotes more resources to the production of ovules than to pollen for all values of the model parameters, i.e., $\hat{\alpha }{>}0.5$ always holds (Proposition 2).
- The optimal allocation devotes an even higher proportion of the resources to ovules if one of the model parameters changes in one of the following ways: if more resources become available ($R\uparrow$), if pollen become less expensive ($r_m\downarrow$), if the pollen dispersal range becomes smaller ($h\downarrow$), or if more pollen is retained ($w\uparrow$) (Proposition 3).

### Compensating for resource decline

The number of fertilized ovules that a population produces is an important measure of the population’s ability to persist in its habitat. A change in any of the determining factors, even from one year to the next, can lead to a reduction in the number of fertilized ovules, perhaps endangering persistence. Among these are environmental factors such as habitat degradation and expanded pollen dispersal range, and evolutionary developments such as larger gametes. In this section, the model will be investigated to determine whether a decline in the gametic production resources *R* can be compensated by adjusting a second model parameter, in order to maintain the number of fertilized ovules that was expected before the environmental change, regardless of whether it equalled the maximum number realizable under the previous conditions or was less than this. The results are of relevance not only for species persistence in their natural ecosystems but also for the management of natural regeneration in forests. Where possible, limits to compensability are derived analytically from the model equations, and results are illustrated graphically for variable model parameters.

Changes in many facets of the environmental conditions may lead to a decline in the resources that are available for gamete production. A few examples of such environmental changes are lower soil moisture, cooler temperatures, or increased shade as the canopy closes. Since for unchanged sexual allocation the number of gametes should depend on the resources available for their production, fewer ovules and pollen are produced. Then, if the pattern of pollen dispersal remains the same, both the expected proportion and thus the expected number of fertilized ovules also decrease. Especially if the resource decline continues over a longer period of time, population persistence depends on the ability of the population to develop modes of reproduction that maintain the number of fertilized ovules and thus to compensate for the resource decline.

**Fig. 3:**
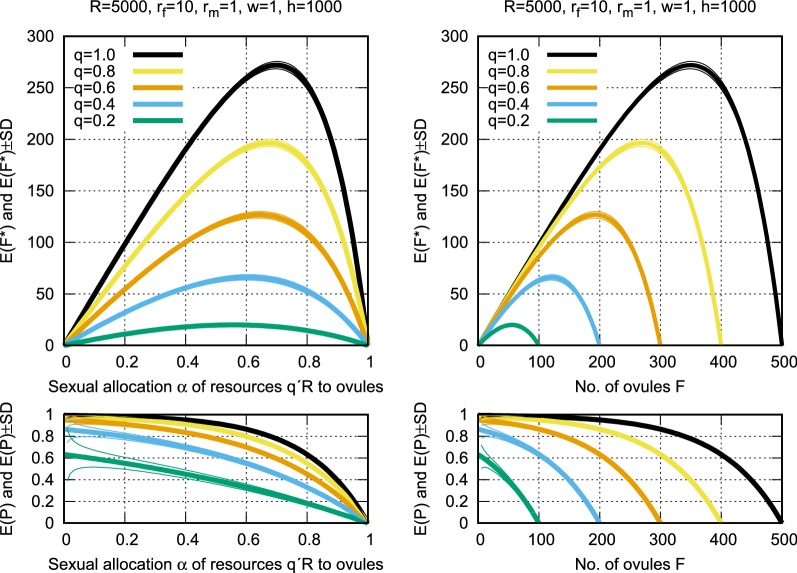
Expected number of fertilized ovules $E(F^*)$ and expected proportion of fertilized ovules *E*(*P*) for full resources $R{=}5000$ and declined resources $q{\cdot }R$ as functions of the sexual allocation $\alpha$ (left panels) and of the number of ovules *F* (right panels). The maximum number of ovules equals $q{\cdot }R/r_f$. The standard deviation $\pm SD$ of $E(F^*)$ is plotted as $F{\cdot }\left( E(P){\pm }\sqrt{V(P)}\right)$

In terms of the model parameters, suppose that the changed environment offers only a proportion $q{\in }(0,1)$ of the resources that were available, say, one or a few years or perhaps a generation ago, that is, the resources are rather suddenly reduced to $q{\cdot }R$. Assuming that the other parameters $r_m$, $r_f$, *w*, *h*, and $\alpha$ are not affected by the environmental change, the population now produces the smaller number $F^\prime = qF$ of ovules and the smaller number $M^\prime {=}\, qM$ of pollen (calculated as $F^\prime = \alpha (q{\cdot }R)/r_f{=}qF$ and $M^\prime = (1{-}\alpha )(q{\cdot }R)/r_m=qM$). The fewer gametes reduce the expected proportion of fertilized ovules to $E(P^\prime )\, =\, 1{-}\left( 1{-}\frac{1}{h}\right) ^{wM^\prime }$ and the expected number of fertilized ovules to $F^\prime {\cdot }E(P^\prime )\, =\, E(F^{\prime *})$. The smaller *q* becomes, the smaller is the expected number of fertilized ovules (see Fig. 3).

Two possible adjustments of the mode of reproduction that could compensate for resource decline are investigated in the following.

### Adjusting sexual allocation

Particularly for monoecious plants, it is commonly observed that female:male function varies among population members over space and time [8, 22, 23]. This variation can be conceived to be the result of differential allocation of resources to the two sexual functions, in which not only genetic but also environmental determinants could be involved. For example, Karlin and Lessard [18] include light exposure, soil composition, moisture, and stress conditions among the latter. While Guo et al. [17] found that allocation to male function increased with mean annual temperature and precipitation, Ejsmond et al. [11] reported a general trend for plants to produce fewer (and larger) pollen grains under higher temperatures and dessication, in which case the number of fertilized ovules might be maintainable by increasing ovule production. Thus the role of sexual allocation of resources in determining fertilization success under variable resource availability is of primary evolutionary relevance. The question to be answered here is whether it might be possible to compensate for resource decline by changing the sexual allocation.

In terms of the model, again quantify the decline in gametic production resources as the proportion *q* of the previous resources *R*. Let $E(F^*)$ denote the expected number of fertilized ovules for resources *R* and the previous allocation $\alpha ^\circ$. If the maximum expected number of fertilized ovules for resources *qR* over the entire range of allocations $\alpha {\in }(0,1)$ is less than $E(F^*)$, then compensation is certainly not possible. This is the case, for example, when the population produced the maximum number of fertilized ovules over all possible allocations in a relatively stable environment before resource decline. This optimal allocation will, however, seldom be realized in populations that are exposed to variable environmental conditions, suggesting that suboptimality of the number of fertilized ovules may be characteristic of frequently changing environments.

Obviously, compensation is impossible if the maximum obtainable number of fertilized ovules falls short of the previously expected number. In formal terms, the reduction in number of fertilized ovules for declined resources *qR* can be compensated by adjustment of the allocation, if it holds that

$$\begin{aligned} E(F^*)\le \max E(F^{*\prime }) \quad \text{ for } \text{ all } \text{ allocations } \alpha {\in }(0,1) \end{aligned}$$

Due to the failure to find an analytical expression for the allocation that corresponds to $\max E(F^{*\prime })$, and thus for the maximum itself, a more exact compensation condition cannot be given here.

It is interesting that for any allocation $\alpha _1$ there exists a second allocation $\alpha _2$ that yields the same expected number of fertilized ovules $E(F^*)$ (see Fig. 2). The sole exception is the optimal allocation $\hat{\alpha }$. Mathematically, this duality of allocations follows from the concavity of the curve $E(F^*)$, which ranges between 0 for the dual allocations to only one gametic sex and the maximum for $\hat{\alpha }$. One of the dual allocations yields a larger number of ovules and a smaller *E*(*P*) and the other fewer ovules and a larger *E*(*P*) which, when multiplied, give the same $E(F^*)$. This phenomenon seems difficult to explain biologically.

**Fig. 4:**
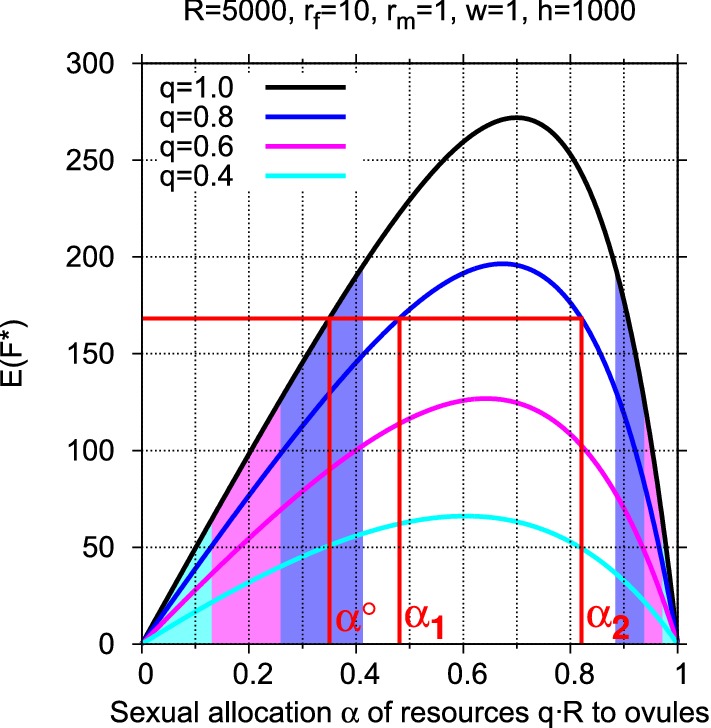
Compensation by adjusting sexual allocation: For parameters $R = 5000$, $r_f = 10$, $r_m = 1$, $w = 1$, $h = 10000$, the colored areas show where adjustment of the sexual allocation $\alpha ^\circ$ can compensate for the loss of fertilized ovules when resources decline to $R = q{\cdot }R$. If $q = 0.8$, for example, and if $\alpha ^\circ$ lies within the range of any of the colors, then there exist two allocations $\alpha _1$ and $\alpha _2$ whose expectation $E(F^{*\prime })$ equals the original expectation $E(F^*)$ for $\alpha ^\circ$. This is illustrated for $\alpha ^\circ = 0.35$ and $q = 0.8$ (red lines). If $q = 0.6$, such compensation is possible only if $\alpha ^\circ$ lies within the magenta or light blue range. If $q = 0.4$, compensation is possible only if $\alpha ^\circ$ lies within the light blue range. Otherwise, compensation is not possible by adjusting the allocation

As Fig. 4 shows, if the previous allocation $\alpha$ to ovules is less than the optimal allocation for the previous resources *R* (i.e., lies within the colored area on the left side), compensation under *qR* can be reached by both a moderate and a considerable increase in the allocation to ovules. Analogously, if $\alpha$ is greater than the optimal allocation (i.e., lies within the colored area on the right side), compensation can be reached by both a moderate and a considerable increase in the allocation to pollen. In terms of the actual numbers of ovules and pollen, more or many more ovules must be produced under *qR* if $\alpha$ is less than the optimal allocation for *R*, in spite of the resource decline. If $\alpha$ is greater than the optimal allocation, more or many more pollen must be produced. The opposite relationship holds for the number of pollen.

### Adjusting pollen density

If fewer pollen are produced as a result of resource decline but the range over which these pollen disperse remains the same, the pollen density sinks and with it the probability that any given ovule will be fertilized. The previous fertilization probability could be maintained if the pollen density were to increase. Changes in wind patterns or the erection of barriers to wind-borne dispersal as a management measure, for example, could reduce the size of the pollen dispersal range (*h*) or increase pollen retention *w* within the range. The larger pollen found by Ejsmond et al. [11] under higher temperatures and desiccation might disperse over shorter distances, thereby increasing pollen retention *w*. But since resource decline also results in fewer ovules, the previous expected number of fertilized ovules can only be maintained if the increased fertilization probability is large enough to also compensate for the smaller number of ovules.

There are, however, limits to the possibilities for compensation by adjusting pollen density. If, for example, the size of the pollen dispersal range is already small, making it smaller will have little effect on the fertilization probability. In like manner, if most of the pollen already remained within the range, retaining an increased proportion will also not be able to increase the fertilization probability sufficiently to yield the previous number of fertilized ovules.

In terms of the model, when resources decline to *qR* for a $q{\in }(0,1)$, the number of ovules is reduced to $q{\cdot }F$ and the number of pollen to $q{\cdot }M$. The expected proportion of fertilized ovules falls from $E(P)=1{-}\left( 1{-}\frac{1}{h}\right) ^{wM}$ to $1{-}\left( 1{-}\frac{1}{h}\right) ^{qwM}$. In order to maintain the previous expected number of fertilized ovules $E(F^*)=F{\cdot }E(P)$, the expected proportion must be raised to $E(P^\prime )=\frac{1}{q}{\cdot }E(P)$. This can only be achieved by increasing *w* or reducing *h*, both of which increase the pollen density within the dispersal range.

**Fig. 5:**
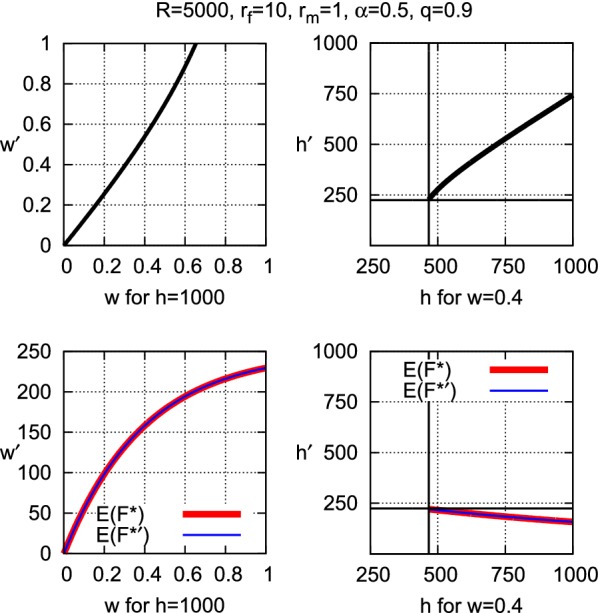
Compensating for reduction of expected number $E(F^*)$ of fertilized ovules when *R* declines to $0.9{\cdot }R$. Left panel: by adjusting *w*. Compensation is not possible for $w{>}0.65$, since $w^\prime = 1$ for $w = 0.65$. Right panel: by adjusting *h*. The smallest size *h* of the pollen dispersal range for which the compensation condition $q{>}E(P)$ is fulfilled equals 435, but the corresponding $h^\prime$ would equal only 71.5 (Proposition 4), which is much less than the number $qF = 225$ of ovules that must lie within the dispersal range. The smallest value of *h* for which $h^\prime {\ge }qF$, and thus for which compensation is possible, equals 466

**Fig. 6:**
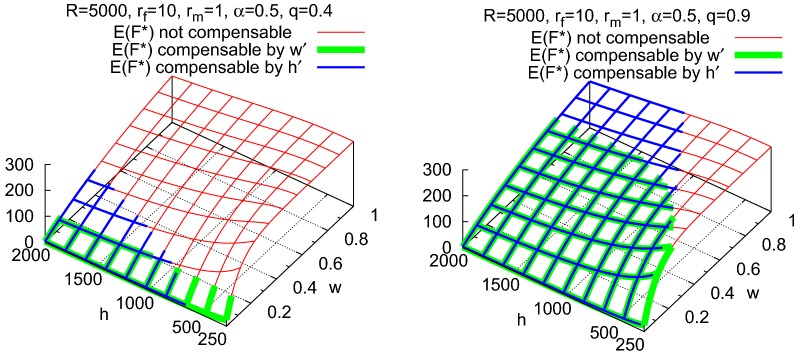
If the gametic production resources *R* decline to the proportion $q{\cdot }R$, the corresponding reduction of fertilized ovules $E(F^*)$ may be compensable in two ways: By adjusting the proportion *w* of retained pollen to $w^\prime$, or by adjusting the size *h* of the pollen dispersal range to $h^\prime$. Left panel: For $q = 0.4$, compensation is possible by adjustment of *w* only for very small *w* (green) and by adjustment of *h* for small *w* and large *h* (blue). Right panel: For $q = 0.9$, compensation is possible by adjustment of *w* for more than half of the (*w*, *h*)-combinations (green) and by adjustment of *h* for most of the (*w*, *h*)-combinations (blue)

Conditions under which compensation is possible by adjusting *w* or *h* are proven in Appendix (Propositions 4 and 5). In particular, adjustment is only possible in either case if the reduction of resources is not too severe, in that the remaining proportion *q* of the resources *R* after reduction is larger than the previous expected proportion *E*(*P*) of fertilized ovules. The retention *w* can be adjusted if it is small enough to be made appreciably larger, and the size of the dispersal range *h* can be adjusted if it is large enough to be made appreciably smaller. The results are illustrated by examples in Figs. 5 and 6. Note that reduction of the size *h* of the pollen dispersal range to a smaller size $h^\prime {<}h$ is only possible within the model framework if all of the *qF* ovules are located within these fewer spatial units and the *wqM* pollen that are retained disperse only to these units. Unless the ovules happen to be clustered within the original pollen dispersal range, this could necessitate the targeted delivery of pollen to the fewer units, as is the case for insect pollination.

## Discussion

Model analysis demonstrates that compensation for resource decline is possible under certain conditions through adjustment of sexual allocation of gametic production resources or pollen density. Possibilities for other parameters are discussed.

### Sexual allocation

The maximum number of fertilized ovules and the corresponding optimal sexual allocation set limits on compensation. An unexpected result of model analysis is that maximization of the expected number $E(F^*)$ of fertilized ovules always requires allocation of a larger proportion of the gamete production resources to ovule production than to pollen production, that is, $\hat{\alpha }{>}0.5$ (Proposition 2).

Suppose that $r_f$ remains unchanged. It follows from Proposition 3 that $\hat{\alpha }$ increases whenever at least one of the following occurs: the resources *R* or the proportion *w* of the pollen that remain within the dispersal range increase, or the investment $r_m$ per pollen or the size *h* of the dispersal range decrease. Conversely, as *R* or *w* decrease, or $r_m$ or *h* increase, the optimal allocation $\hat{\alpha }$ decreases toward 0.5.

A practical consequence of this observation is the prediction that populations in habitats with abundant resources may be found to devote a much larger proportion of their resources to ovule production than to pollen, while populations in poor environments will devote an increasingly even proportion—up to one-half—of their meager resources to pollen production. Unfortunately, such a shift in the sexual allocation of resources towards pollen production may not be able to raise the number of fertilized ovules in the poor environment to that in a good environment.

As an example, Fig. 3 shows that as the resources *R* decline from 5000 ($q = 1.0$) to 1000 ($q = 0.2$), the optimal allocation to ovules decreases from $\hat{\alpha }= 0.699$ to 0.557. The result of this shift toward increased pollen production is a drastic drop of the maximum expected number $\max E(F^*)$ of fertilized ovules from 272 to a mere 20. This emphasizes the sensitivity of a population’s chances for persistence to changes in the available resources.

The model also suggests that if resources remain unchanged, it is not advisable to devote more than the optimal allocation to ovules. Since the slope of the curve $E(F^*)$ is steep for $\alpha {>}\hat{\alpha }$, especially for abundant resources, production of an excess of ovules can sharply reduce the expected number of fertilized ovules. The detrimental effect on $E(F^*)$ is less pronounced if the allocation remains slightly under $\hat{\alpha }$ than if it exceeds $\hat{\alpha }$ by the same amount.

The frequently observed variability of sexual allocation to ovule and pollen production within monoecious plant species suggests that compensation may actually occur in this way ([7]). The prerequisite is that the resource decline is not too severe, in that the expected number $E(F^*)$ of fertilized ovules for the allocation before the decline is not less than the new optimum $\max E(F^{*\prime })$ over all allocations after the decline. In all such cases, two different allocations, one on either side of the new optimum, dually provide compensation. In effect, the possibility of compensation by dual allocations implies that pollen can be substituted by ovules and *vice versa*. Beyond the mathematical properties of the model that produce this effect, it is interesting to speculate on the mechanisms behind this substitutability.

### Pollen density

Compensation is also possible by adjusting the pollen density $d{=}wM/h$, but only if the resource decline is bounded by a limitation that is non-intuitive, namely that the proportion *q* of resources that remain exceeds the expected proportion *E*(*P*) of fertilized ovules. Compensation requires that the reduction in the numbers of produced gametes caused by resource decline can be counterbalanced by an increase in pollen density in spite of the fewer pollen. This can succeed by increasing the pollen retention if the original retention *w* was small or by shrinking the dispersal range from its original size *h* so that it still contains all of the ovules. Especially in wind-pollinated species, both parameters *w* and *h* heavily depend on the meteorological conditions. Since the resources may also depend on the same meteorological conditions to some degree, the supposed independence in the model between the resource parameter *R* and the parameters *w* and *h* may not be realistic. This consideration suggests that the study of the interdependence between resource availability and patterns of pollen dispersal and retention could aid in the prediction of persistence in natural populations.

### Other parameters

It is conceivable that a population might also be able to compensate for resource decline by reducing the investment per ovule or pollen. By this, the reduction in the number of gametes caused by resource decline could be at least partially offset by devoting the remaining resources to the production of more “less expensive” ovules or pollen (sex allocation theory of Charnov [5]). Modeling such an adjustment is more complex than a simple change in model parameters, however. Reducing the investment per gamete would not only require genetic changes that may only be realizable on a longer-term evolutionary scale. It could also affect the overall fertilization probability by lowering the capacity of individual pollen or ovules for fertilization ([9]), so that the model assumption that an ovule is fertilized when at least one pollen lands on it is no longer sufficient. Analysis of these aspects requires additional efforts that are beyond the present scope.

## Conclusion

The results have a number of implications, some of which are intuitively apparent and others may be difficult to comprehend. A few examples are briefly addressed.

### Application to insect-pollination

The model was formulated with wind-pollinated plants in mind, especially the assumption of random pollen dispersal, but it may also be made applicable to insect-pollinated species by adjusting the relevant parameters. Since pollen is transported directly to ovules, pollination by insects may have a large effect on the shape and size of the pollen dispersal range. In effect, the pollen dispersal range is almost restricted to those spatial units that actually contain an ovule, since insects are unlikely to deposit pollen in empty units. Thus the size *h* of the range would be close to the number *F* of ovules produced and thus be as small as possible. On the other hand, insects may not collect all of the pollen that a flower produces and then feed some the collected pollen to their larvae, making the proportion *w* of pollen that actually reach the ovules small. As a result of both considerations, the central variable *b* for optimization of fertilization success is indeterminate with the result that the final effect may be comparable to situations typical for wind pollination. This example demonstrates the versatility of the model and shows that even apparently extreme cases can show “average” behavior.

### Bias of sexual allocation

Sexual allocation in natural populations that are subject to changing environmental conditions, and thus varying resources, is presumably rarely optimal. Yet, knowledge of the optimum in each of the conditions is essential in order to assess the “reproductive stress” to which a population is exposed under its current condition. For this reason, some effort was taken to characterize the optimum sexual allocation. A particularly conspicuous result concerns the bias of the optimum towards ovule production ($\hat{\alpha }{>}0.5$), irrespective of the model parameters and especially of the investment per gametic type. This pervasive asymmetry calls for a corresponding asymmetry in the model assumptions that does not depend on the parameter values. The assumption of random dispersal of pollen over the spatial units cannot be held responsible, since in effect it is equivalent to random dispersal of ovules over the units. The difference lies in the assignment of ovules and pollen to spatial units, in that each unit can be occupied by at most one ovule but by arbitrarily many pollen. In terms of sampling methods, this corresponds to the sampling of spatial units without replacement for assignment to ovules and the sampling of spatial units with replacement for assignment to pollen. The mathematical proofs confirm that this characteristic of the model imposes a bias on the optimal sexual allocation towards ovule production. An intuitive explanation for this bias seems elusive.

### Management for compensation

The maintenance of the expected number of fertilized ovules in the face of resource decline is an important goal for the conservation or sustainable management of populations. The resource manager could attempt to maintain the number of fertilized ovules that was expected before the resource decline by adjusting a single characteristic of reproduction. As the model reveals, adjustment of the sexual allocation $\alpha$ would only be successful if the expected number of fertilized ovules before the decline is not greater than the number attainable under optimal sexual allocation after the decline. Adjustment of the pollen density could be successful only if the proportion of resources that remain is larger than the expected proportion of fertilized ovules before the decline. In this case, increasing the pollen density by reducing the size *h* of the dispersal range would be successful only if this range was not already too small. Increasing the pollen density by increasing the pollen retention *w* would work only if the original value lay within an intermediate range: if the retention was too high, it could not be increased enough to help, and if retention was too low, an increase would not suffice. Whenever compensation is not possible, the expected number of fertilized ovules decreases, and with it the chances that the population can persist under the reduced resources. Practical measures to implement compensation depend in many ways on the reproduction characteristics of each species. Even an attempt to outline appropriate methods would however exceed the scope of the present paper.

### Metapopulation fragmentation

When the individual population is considered as part of a metapopulation, the destination of pollen that does not remain within the dispersal range becomes relevant. Such pollen may contribute to neighboring populations or it may become lost due to isolation between the parts, as is typical for fragmentation. Thus increasing $(1{-}w)$ in fragmented (meta)populations destabilizes these via reduction of fertilization opportunities. Of the many model parameters that can be used to characterize destabilizing effects of fragmentation, *w* is probably the most efficient.

## Figures and Tables

**Fig. 7:**
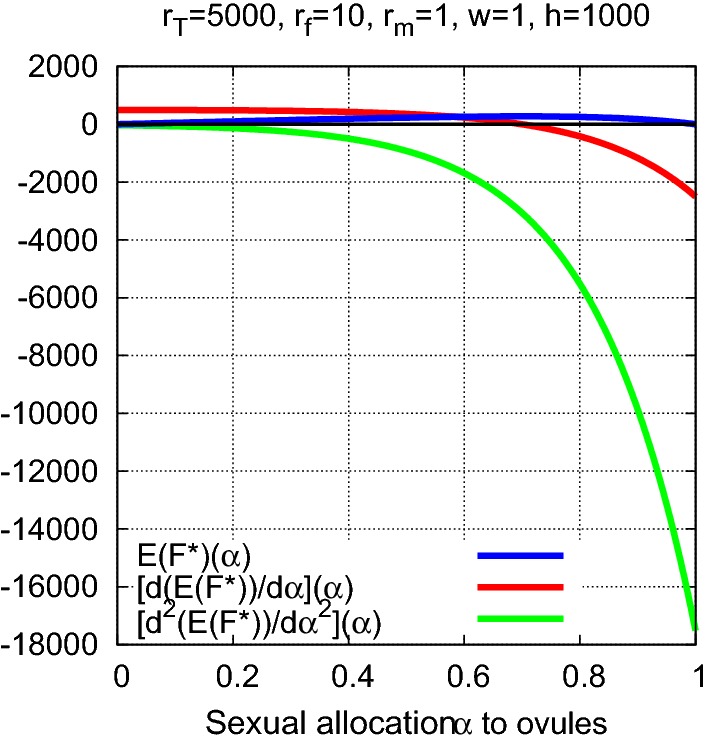
The expected number of fertilized ovules $E(F^*)$ as a function of the sexual allocation $\alpha$ of resources to ovules, together with its first and second derivatives, for $R{=}5000$, $r_f{=}10$, $r_m{=}1$, $w{=}1$, and $h{=}1000$

**Fig. 8:**
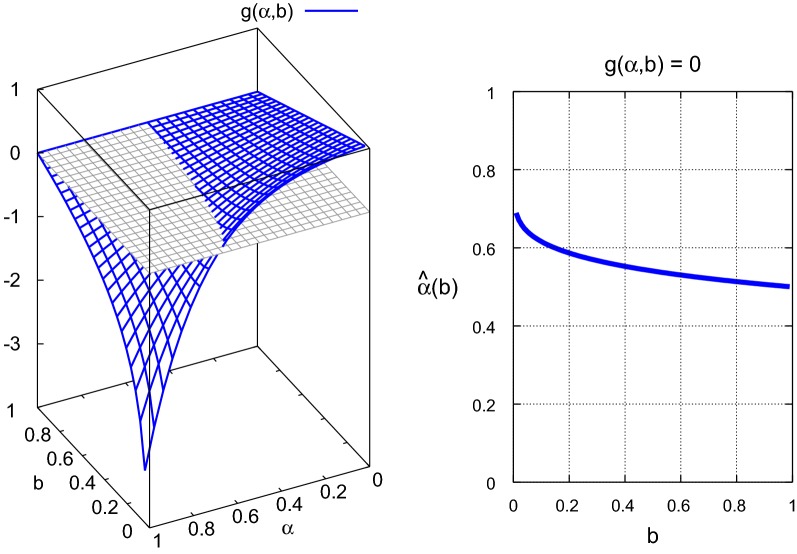
The surface $g(\alpha ,b)=b^{1{-}\alpha }{\cdot }\big (\alpha {\cdot }\ln (b){-}1\big ){+}1$ (blue) is plotted as a function of $\alpha$ and *b* and intersected by the plane at 0 (blue) (left panel). The curve $\hat{\alpha }(b)$ as the implicit function $g(\alpha ,b){=}0$ is plotted as the intersection of the surface $g(\alpha ,b)$ with the plane, demonstrating that $\hat{\alpha }(b){>}0.5$ for all *b* and that $\hat{\alpha }$ is a strictly decreasing function of *b* for $0{<}b{<}1$ (right panel)

**Fig. 9:**
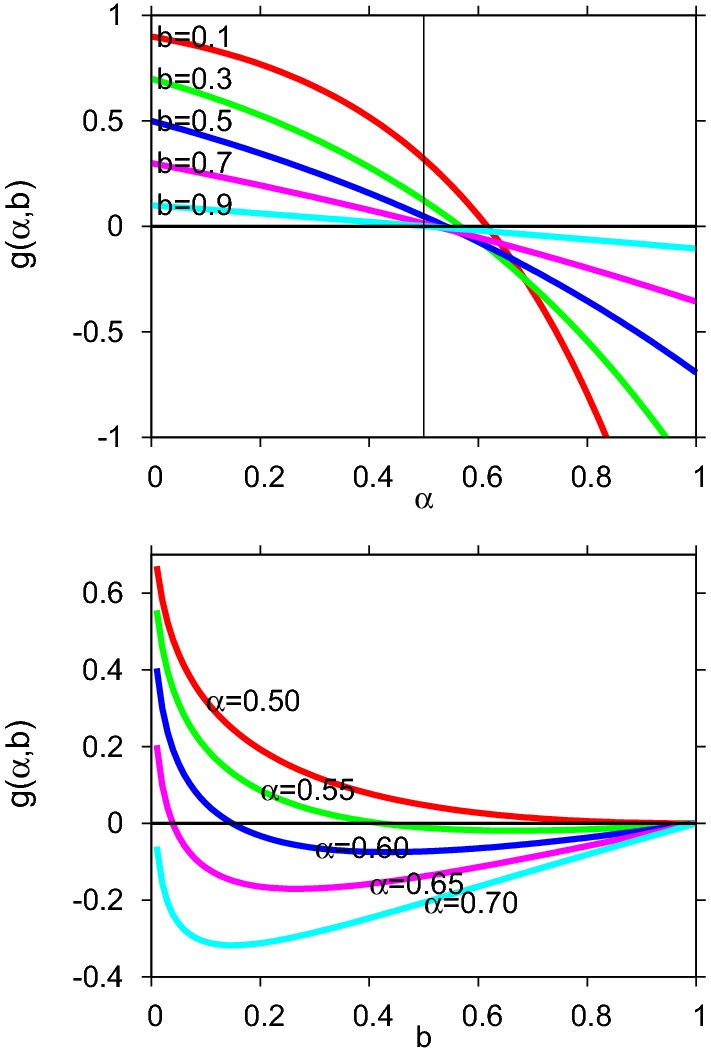
Curves $g(\alpha ,b)=b^{1{-}\alpha }\left( \alpha \ln (b){-}1\right) +1$ as functions of $\alpha$ for different values of *b* (upper panel) and as functions of *b* for different values of $\alpha$ (lower panel). For given *b*, the optimal sexual allocation $\hat{\alpha }$ is the value of $\alpha$ for which $g(\alpha ,b)$ equals 0. Whereas $\hat{\alpha }$ exists for every $b{\in }(0,1)$ and is greater than 0.5, only for $\alpha {>}0.5$ is there a *b* for which $g(\alpha ,b){=}0$. The optimum $\hat{\alpha }$ increases as *b* decreases

## Data Availability

No real data is used. All data sets used to generate the figures are specified in the figures or their legends. Data sets referred to in the Results section are specified in the text. If additional clarification is needed, please contact the corresponding author.

## Appendix

### **Proposition 1**

$E(F^*)$*assumes its maximum for a single allocation*$\hat{\alpha }$*to ovules. Defining*$b{=}\left( 1{-}\frac{1}{h}\right) ^{wR/r_m}$, *this optimal allocation*$\hat{\alpha }$*is implicitly expressed by the equation*

$$\begin{aligned} b^{1{-}\hat{\alpha }}\left( \hat{\alpha }\ln (b)-1\right) +1=0 \end{aligned}$$

### *Proof*

Express the function $E(F^*)$ and its first two partial derivatives with respect to $\alpha$ using $b{=}\left( 1{-}\frac{1}{h}\right) ^{wR/r_m}$ as

$$\begin{aligned} E(F^*)=\, & {} \left( \alpha {\cdot }\frac{R}{r_f}\right) \cdot \left( 1{-}b^{1{-}\alpha }\right) \\ \frac{\partial }{\partial \alpha }\left[ E(F^*)\right]=\, & {} \frac{R}{r_f}\left[ b^{1{-}\alpha }\left( \alpha \ln (b){-}1\right) +1\right] \\ \frac{\partial ^2}{\partial \alpha ^2}\left[ E(F^*)\right]= & {} -\frac{R}{r_f}\left[ b^{1{-}\alpha }\ln \left( b\right) \left( \alpha \ln (b){-}2\right) \right] <0 \end{aligned}$$

(see Fig. 7). Because $\frac{\partial }{\partial \alpha }\left[ E(F^*)\right] = \frac{R}{r_f}\left( 1{-}b^{1{-}\alpha }\right) >0$ for $\alpha {=}0$, $\frac{\partial }{\partial \alpha }\left[ E(F^*)\right] =\frac{R}{r_f}{\cdot }\left( \alpha \ln (b)\right) <0$ for $\alpha {=}1$, and $\frac{\partial ^2}{\partial \alpha ^2}\left[ E(F^*)\right]$ is a decreasing function of $\alpha {\in }[0,1]$, the single allocation $\hat{\alpha }$ for which $\frac{\partial }{\partial \alpha }\left[ E(F^*)\right] =0$ holds maximizes $E(F^*)$. For lack of a closed-form expression for the optimal allocation $\hat{\alpha }$, the requirement that $\frac{\partial }{\partial \alpha }\left[ E(F^*)\right] {=}0$ holds yields the implicit expression $b^{1{-}\hat{\alpha }}\left( \hat{\alpha }\ln (b)-1\right) +1=0$ (see Figs. 8 and 9). $\square$

### **Proposition 2**

*The optimal allocation*$\hat{\alpha }$*that maximizes the expected number of fertilized ovules*$E(F^*)$*devotes more resources to the production of ovules than to pollen (i.e.,*$\hat{\alpha }{>}0.5$) *for all values of the model parameters.*

### *Proof*

As shown above, the optimal allocation $\hat{\alpha }$ solves $b^{1{-}\hat{\alpha }}\left( \hat{\alpha }\ln (b)-1\right) +1{=}0$. Define

$$\begin{aligned} g(\alpha ,b)=b^{1{-}\alpha }\cdot \left( \alpha {\cdot }\ln (b)-1\right) +1 \end{aligned}$$

as a function of variables $\alpha$ and *b*. For any given *b*, *g* is a strictly decreasing function of $\alpha$. In particular, $g(0,b){=}1{-}b{>}0$ and $g(1,b){=}\ln (b){<}0$, so that for each $b{\in }(0,1)$ there exists exactly one $\alpha$ for which $g(\alpha ,b)=0$. Therefore, $\hat{\alpha }(b)$ is a proper function of *b* that is implicitly defined by $g(\alpha ,b)=0$. In particular, $g(0.5,b)=b^{0.5}{\cdot }\left( 0.5{\cdot }\ln (b){-}1\right) +1=b^{0.5}{\cdot } \left( \ln (b^{0.5}){-}1\right) +1$. Hence, if $g(0.5,b){>}0$ then the solution of $g(\alpha ,b)=0$ for $\alpha$ necessarily entails $\alpha {>}0.5$ and thus $\hat{\alpha }(b){>}0.5$. Since $x\cdot \left( \ln (x)-1\right) +1$ is a strictly decreasing function of *x* with minimum 0 for $x{=}1$, the solution of $g(\alpha ,b){=}0$ indeed always yields $\alpha {=}\hat{\alpha }(b){>}0.5$. $\square$

### **Proposition 3**

$\hat{\alpha }(b)$*is a strictly decreasing function of**b**for*$0{<}b{<}1$.

### *Proof*

Recall that by the rule of taking derivatives of implicitly defined functions one has $d\hat{\alpha }(b)/db=-(\partial g/\partial b)/(\partial g/\partial \alpha )$. Herewith, $\partial g/\partial \alpha {<}0$ as is shown above, so that $d\hat{\alpha }(b)/db<0$ if $\partial g/\partial b<0$. Straightforward calculations yield

$$\begin{aligned} \partial g/\partial b= b^{-\alpha }\cdot \big [(1{-}\alpha )\cdot (\alpha {\cdot }\ln (b)-1)+\alpha \big ] \end{aligned}$$

where the term in square brackets strictly increases with *b* for any given value of $\alpha {\in }(0,1)$. Consider $\partial g/\partial b$ to be the function $u(\alpha ,b)=b^{-\alpha } \cdot \big [(1{-}\alpha )\cdot (\alpha {\cdot }\ln (b)-1)+\alpha \big ]$ and recall that $b^{-\alpha }{>}0$. Suppose that $u(\hat{\alpha }(b),b){\ge }0$ for some *b*. Then $u(\hat{\alpha }(b),b^\prime ){>}0$ for all $b^\prime {>}b$. Consequently, $g(\hat{\alpha }(b),b^\prime )$ increases with $b^\prime {>}b$, and it is positive due to $g(\hat{\alpha }(b),b){=}0$. Thus $g(\hat{\alpha }(b),1){>}0$, which contradicts $g(\alpha ,1){=}0$ for all $\alpha$. Therefore, $u(\hat{\alpha }(b),b){<}0$ with the result that $\hat{\alpha }(b)$ decreases strictly with increasing *b*. $\square$

### **Proposition 4**

*For given**R*, $r_f$, $r_m$, $\alpha$, *w*, *and*$h{\ge }F{=}\alpha R/r_f$, *suppose that**R**declines to*$q{\cdot }R$*for*$q{\in }(0,1)$. *For unchanged pollen dispersal range**h*, *the reduction of*$E(F^*)=F\cdot \left( 1{-}\left( 1{-}\frac{1}{h}\right) ^{wM}\right)$*to*$E(F^{\prime *}) =qF\cdot \left( 1{-}\left( 1{-}\frac{1}{h}\right) ^{qwM}\right)$*can be compensated by increasing the pollen retention from**w**to*

$$\begin{aligned} w^\prime =\, \frac{\ln \left( 1{-}\frac{1}{q} \left( 1{-}\left( 1{-}\frac{1}{h}\right) ^{wM}\right) \right) }{qM{\cdot }\ln \left( 1{-}\frac{1}{h}\right) } \end{aligned}$$

*under the conditions that*$q{>}\left( 1{-}\left( 1{-}\frac{1}{h}\right) ^{wM}\right) =\, E(P)$*holds for*$M = (1{-}\alpha )R/r_m$, *or equivalently*$w>\displaystyle \frac{\ln (1{-}q)}{M\ln \left( 1{-}\frac{1}{h}\right) }$, *and if it holds that*

$$\begin{aligned} w\le \frac{\ln \left( 1{-}q\left( 1{-} \left( 1{-}\frac{1}{h}\right) ^{qM}\right) \right) }{M\ln \left( 1{-}\frac{1}{h}\right) } \end{aligned}$$

### *Proof*

The resource decline reduces the number of ovules and pollen to *qF* and *qM*, respectively. In order to maintain the previous expected number of fertilized ovules, find $w^\prime$ in the interval (0, 1] for which it holds that $E(F^*)=F\cdot \left( 1{-}\left( 1{-}\frac{1}{h}\right) ^{wM}\right) = qF\cdot \left( 1{-}\left( 1{-}\frac{1}{h}\right) ^{w^\prime qM}\right)$. Solving this equation yields the above expression for $w^\prime$, but this expression is defined and greater than 0 only if the operand of the logarithm in the numerator is greater than 0, i.e., if $q>\left( 1{-}\left( 1{-}\frac{1}{h}\right) ^{wM}\right)$. This inequality also places a lower bound on the retention parameter *w* for which compensation is possible as $w>\displaystyle \frac{\ln (1{-}q)}{M\ln \left( 1{-}\frac{1}{h}\right) }$. Solving the equation $w^\prime \le 1$ for *w* yields the upper bound of the parameter *w* for which compensation is possible. $\square$

### **Proposition 5**

*For given**R*, $r_f$, $r_m$, $\alpha$, *w*, *and*$h{\ge }F{=}\alpha R/r_f$, *suppose that**R**declines to*$q{\cdot }R$*for*$q{\in }(0,1)$. *For unchanged pollen retention**w*, *the reduction of*$E(F^*)$*to*$E(F^{\prime *})$*can be compensated by decreasing the size of the dispersal range from**h**to*

$$\begin{aligned} h^\prime =\displaystyle \frac{1}{1{-}\left[ 1{-}\frac{1}{q} \left( 1{-}\left( 1{-}\frac{1}{h}\right) ^{wM}\right) \right] ^{1/(qwM)}} \end{aligned}$$

*under the conditions that*$q{>}\left( 1{-}\left( 1{-}\frac{1}{h}\right) ^{wM}\right) = E(P)$*holds for*$M = (1{-}\alpha )R/r_m$, *or equivalently, that the pollen dispersal range**h**fulfills*

$$\begin{aligned} h>\displaystyle \frac{1}{1{-}(1{-}q)^{1/(wM)}} \end{aligned}$$

*and that the smaller dispersal range of size*$h^\prime$*within which**wqM**pollen are retained contains all of the**qF**ovules, implying that*$h^\prime \ge qF$.

### *Proof*

For the reduced number of ovules *qF* and pollen *qM*, find $h^\prime >0$ for which it holds that $E(F^*)=F\cdot \left( 1{-}\left( 1{-}\frac{1}{h}\right) ^{wM}\right) = qF\cdot \left( 1{-}\left( 1{-}\frac{1}{h^\prime }\right) ^{wq M}\right)$. Solving this equation yields the above expression for $h^\prime$, but this expression is defined only if the denominator is defined, that is, if $q>\left( 1{-}\left( 1{-}\frac{1}{h}\right) ^{wM}\right)$. This inequality is equivalent to the lower bound for *h* given above and also ensures that $h^\prime \ge 1$ holds. The smaller dispersal range of size $h^\prime$ must contain all of the *qF* ovules. Since each spatial unit can be occupied by at most one ovule, $h^\prime$ cannot be smaller than *qF*. $\square$
